# The effect of breast massage combined with co-parenting interventions on breastfeeding in mother-infant separated mothers: a quasi-experimental study

**DOI:** 10.3389/fnut.2026.1770798

**Published:** 2026-04-22

**Authors:** Rong Li Zhang, Yi Jing Wang, Ying Yin, Ying Liu, Ping Cui, Zhang Yi Lu, Li Jun Deng, Hui Jiang, Rong Huang

**Affiliations:** Shanghai Key Laboratory of Maternal Fetal Medicine, Shanghai Institute of Maternal-Fetal Medicine and Gynecologic Oncology, Shanghai First Maternity and Infant Hospital, School of Medicine, Tongji University, Shanghai, China

**Keywords:** exclusive breastfeeding, lactation support, mother-infant separation, nutritional outcomes, partner involvement, postpartum depression, postpartum mothers

## Abstract

**Background:**

Exclusive breastfeeding is the optimal source of infant nutrition. Mother-infant separation poses a critical barrier to establishing and sustaining lactation, increasing the risk of early breastfeeding cessation. While breast massage supports milk production, the role of partner involvement in this context remains underexplored as a strategy to improve both nutritional and psychosocial outcomes.

**Objective:**

This study aimed to evaluate the effect of a co-parenting intervention on exclusive breastfeeding rates and related maternal outcomes among postpartum mothers separated from newborns, in which fathers were taught and performed structured breast massage.

**Methods:**

A quasi-experimental study was carried out on 120 mother-infant separated dyads in a tertiary maternity hospital. Participants were assigned to an intervention group (*n* = 60) and a control group (*n* = 60) via consecutive sampling plus baseline characteristic matching (maternal age, mode of delivery, and newborn birth weight). The intervention group received father-performed integrated breast massage training and practice; the control group received routine care. The primary outcome was the exclusive breastfeeding rate, and the secondary outcomes included the scores of the Breastfeeding Self-Efficacy Scale (BSES), breastfeeding knowledge, and the Edinburgh Postnatal Depression Scale (EPDS). All indicators were assessed at baseline, 1 month, and 3 months after the intervention.

**Results:**

At both follow-ups, the intervention group demonstrated significantly higher exclusive breastfeeding rates compared to the control group (1-month, 64.9% vs. 22.4%, 3-months, 64.3% vs. 16.7%, both *p* < 0.05). The intervention group also showed significantly greater improvements in breastfeeding self-efficacy and knowledge scores, and a more significant reduction in EPDS scores over time (all *p* < 0.05). In contrast, the control group exhibited no significant changes in these secondary outcomes across the three time points. No significant inter-group difference was detected in perceived social support.

**Conclusion:**

Integrating fathers into a structured breast massage protocol is an effective, sustainable nutritional support strategy. Structured breast massage significantly enhances exclusive breastfeeding rates, maternal confidence, and mental well-being among separated mothers. These findings advocate for the inclusion of partners in clinical lactation support programs to safeguard optimal infant nutrition and postpartum recovery.

## 1 Introduction

Breast milk provides the nutrients needed for infant growth and is the cornerstone of infant survival and development. The World Health Organization recommends exclusive breastfeeding for 6 months and breastfeeding for 2 years after delivery (1). Breastfed infants have better physical and mental health and are less likely to suffer from diarrhea, pneumonia, obesity and diabetes in later life (2, 3). Benefits to mothers include psychological upliftment, enhanced bonding and reduced incidence of gynecological disorders, including breast and ovarian cancer (4). Exclusive breastfeeding is defined as feeding the infant only breast milk without any other fluids or foods, vitamin/mineral drops or medications (5). In most developed and developing countries, the exclusive breastfeeding rate at 6 months has not reached the desired goal despite tremendous efforts. These countries include the United States (34.70%), China (20.80%), India (58.00%), Pakistan (47.47%) and Kazakhstan (37.77%) (6). This is far from the global target of 50% exclusive breastfeeding rate within 6 months by 2025 (7). One of the most important reasons for the low rate of exclusive breastfeeding is the lack of family support, especially from fathers.

The process of lactation is a complex physiological process with involvement from multiple endocrine systems. Suckling of the newborn or effective stimulation of the nipple sends sensory signals to the hypothalamus, which accelerates and pulses the release of pituitary lactogenic hormones, promoting milk secretion and increasing the breastfeeding rate (8). However, after delivery, the physiology and psychology of the mother are in a relatively fragile stage, and many factors such as nutrition, health and mental status will affect the quality and quantity of breast milk secretion. In particular, depressed mothers lack confidence in breastfeeding, do not actively stimulate the nipples, have insufficient milk secretion to establish effective breastfeeding, and both mothers and infants are in a state of dissatisfaction or difficulty with the breastfeeding process. Some studies have shown that the most common reasons for cessation of breastfeeding are difficulty in sucking rupture and/or tenderness, breast pain and breast swelling. The most common breastfeeding problems include breast pain, inadequate milk supply (perceived or actual), ductal obstruction, swelling, mastitis and breast abscess (9). Current treatments used for the above breastfeeding problems include painkillers, antibiotics, surgical drainage and ultrasound of the affected breast, cold packs, cabbage leaves and various ointments (10). Overall, there is insufficient evidence to recommend any particular treatment; however, treatments to relieve the mother’s discomfort may still have an impact on preventing premature cessation of breastfeeding (11).

Breast massage, a new technique in obstetrics, is a method used to induce the body to produce endogenous prolactin and oxytocin by stimulating the breasts in order to promote milk secretion and reduce maternal breast discomfort. Correctly performed breast massage and effective breast stimulation can lead to reflex secretion of prolactin, help the mammary ducts to become smooth, promote early milk secretion and increase the amount of milk, which are conducive to infant feeding and improve the rate of exclusive breastfeeding (12). For mothers and infants separated after delivery, timely breast massage to promote lactation when the newborn is not with the mother is of importance for breastfeeding. However, at present, China lacks national standards for lactation technology. The lack of industry standards leads practitioners to develop methods of their own. Lactation care effect is therefore variable, the techniques may not be appropriate. If the swollen breasts are squeezed after maternal pain is obvious, acute mastitis may develop, which also causes a decrease in the secretion of breast milk, or may even stop the secretion of milk. This not only increases maternal pain, but also delays the onset of lactation beyond the optimal time.

Current massage techniques include: gua sha therapy, which is a type of massage that consists of short, soft scrapes of the breast tissue to stimulate blood production and improved fluid dissemination (13); Oketani massage, which consists of eight hand techniques, seven techniques for separation of the breast space, and one technique for milking (14); and therapeutic breast massage for lactation, which focuses on gentle massage of the axillae, alternating with gentle breast massage (15). Various researchers have investigated massage techniques without mentioning the specific types of massage. In these studies, massage was described as general hand movements, pushing the breast from the bottom in a circular pattern, and tactile stimulation of the mammary glands and tissues. Massage was also described as using hand movements to roll the knuckles down onto the breast, starting at the ribs and working toward the areola.

The benefits of integrated breast massage techniques are: first, the addition of a final step that applies direct pressure to the breast mass, which improves milk drainage, and second, enhancing the step aimed at improving lymphatic and blood circulation to promote dilation of the milk ducts. Witt et al. found that the optimal duration of massage is 30 min (16).

Based on existing techniques, the integrated breast massage [IBM] (15) in this study is an optimized and standardized intervention that integrates the advantages of current methods and addresses the limitations. Unlike single-technique massage, IBM combines axillary circulation promotion from therapeutic lactation massage, interstitial tissue separation from Oketani massage, and local stimulation from gua sha therapy with an added key step to enhance lymphatic and blood circulation for mammary duct dilatation. Furthermore, IBM standardizes operation intensity (limited to skin and superficial fascia) and body position (supine with 45° head elevation) to prevent iatrogenic injury. The 30-min procedure matches the optimal duration reported in the literature. The standardized protocol resolves clinical practice inconsistencies caused by the lack of national standards in China, facilitating clinical application and implementation by non-professional caregivers, such as fathers.

The roles of and interactions between mother and father during the transition to parenthood are referred to as co-parenting (17). Co-parenting has typically been used to refer to married or divorced couples sharing parenting responsibilities, and during co-parenting, partners can also provide mothers with tools and emotional support during the postpartum period (18). One study found that co-parenting interventions improved co-parenting support, couple communication and parent–child interaction, and alleviated mother’s depressive symptoms (19). Including fathers as members of the co-parenting team improves father’s breastfeeding knowledge, improves father’s attitudes and involvement in breastfeeding, and increases breastfeeding rates (20). Positive co-parenting has been defined as the sharing of responsibilities and goals, as well as teamwork between two individuals who work together to ensure the healthy development of the child (21). Several studies have shown that co-parenting support reduces the level of maternal postpartum depression (22). Another study showed that low levels of co-parenting increased parenting stress (23). Therefore, examining symptoms of depression, anxiety and stress associated with co-parenting may help to enhance the understanding of maternal stress during the perinatal period. Breastfeeding co-parenting describes how parents share their parenting tasks and collaborate to achieve their breastfeeding goals. The breastfeeding co-parenting framework was developed by Abbass et al. and consists of five elements: shared breastfeeding goal setting, shared responsibility for breastfeeding, positive breastfeeding support, father/partner parent–child interaction, and productive communication and problem solving (24).

## 2 Materials and methods

### 2.1 Study design and participants

The current study was a quasi-experimental study conducted in the Obstetric Unit of Shanghai First Maternity and Infant Hospital (Tongji University, Shanghai, China) from October–December 2024. Shanghai First Maternity and Infant Hospital is a teaching hospital accredited as an “AAA” tertiary care specialty hospital. This hospital has a high birth rate with an average of 15,000–18,000 new births annually. Greater than 85% of the pregnant women who give birth in the hospital are local residents and the remaining 10–15% come from other cities. Newborns that are severely premature or have other abnormalities requiring admission to the Neonatal Department for treatment are separated from their mothers during this period.

In this study a non-random group allocation method combining consecutive sampling with baseline characteristics (maternal age, mode of delivery, and newborn birth weight) matching was adopted (25). The entire process of sampling and group allocation was independently conducted by two uniformly trained researchers, while another senior researcher was responsible for quality control to ensure the continuity of sampling and the accuracy of group matching, thereby reducing human operational bias.

#### 2.1.1 Reason for non-random allocation

This study was conducted in a clinical setting of a tertiary maternity hospital. The allocation of participants was restricted by the actual clinical nursing workflow and the voluntary matching of mother-infant separated dyads with the intervention measures. To ensure the continuity of clinical care and avoid interference with the routine nursing work of the obstetric ward, a random number table method could not be used for strict random grouping. Instead, a consecutive sampling method combined with group matching according to baseline characteristics was adopted for grouping. Eligible participants were recruited consecutively according to the admission time and the intervention and control groups were matched one-by-one according to key baseline characteristics (maternal age, mode of delivery, and newborn birth weight) to ensure the balance of baseline data between the two groups, which reduced the selection bias caused by non-random grouping as much as possible.

Mothers were screened for eligibility by an independent researcher when participants were admitted to the Obstetric Unit after delivery. Eligible participants were mothers who met the following criteria: voluntary agreement to participate in the study; accompanied by their father of the baby; capable of reading, writing, and communicating in Chinese; absence of severe obstetric complications or other medical conditions (permanent or temporary) that could affect breastfeeding (e.g., postpartum hemorrhage, acute hepatitis, HIV-positive status, or psychiatric illness); newborn admitted to the Pediatric Department within 24 h postpartum; singleton delivery; voluntary participation of the father of the baby; and willingness to breastfeed. The acceptability of breast massage to the mother and partner was assessed by a linear scale method with three levels: fully acceptable, average and unacceptable. Only those couples who fully accepted breast massage were included in this study. Finally, a total of 120 mother-infant separated dyads were enrolled with 60 in the intervention group and 60 in the control group.

### 2.2 Ethical consideration

The current study was approved by the Ethics Committee of Shanghai First Maternity and Infant Hospital (ethical code: KS24395). The registration number of the Chinese Clinical Trial Center is ChiCTR2500107585. Participants were informed about the purpose and content of the intervention, and the risk and benefit of participation. In addition, the participants were assured of the confidentiality and anonymity of the clinical data. Written informed consent was signed by all participants.

### 2.3 Intervention programs

#### 2.3.1 Control group

Routine nursing care for mother-infant separated mothers at Shanghai First Maternity and Infant Hospital includes health education on the benefits of breastfeeding, techniques and frequency of breast massage (breast massage twice a day for 30 min each time, involving simple circular kneading of the breast parenchyma with gentle circular pressure from the breast base toward the areola, and nipple stimulation by lightly pinching and stretching the nipple with the thumb and index finger to induce the milk let-down reflex), instructions on the use and frequency of medical-grade breast pumps, and kangaroo mother care for separated newborns. Fathers were present throughout the entire health education process.

Routine breast massage for the control group was primarily performed by on-duty obstetric nurses in the ward; newborn fathers could participate on a voluntary basis.

The massage was conducted twice a day for 30 min each time until mother-infant reunion with no fixed body position (mothers could choose a supine or sitting position for comfort).

A total of 11 fathers (18.33%) in the control group voluntarily participated in the mothers’ breast massage. Among the fathers, only 3 participated in > 50% of the massage sessions, 5 participated in 20–50% of the sessions, and 3 participated occasionally in 1 or 2 sessions.

#### 2.3.2 Intervention group

The intervention group adopted IBM based on the same routine nursing care for mother-infant separated mothers as the control group at Shanghai First Maternity and Infant Hospital (15), instead of the conventional breast massage technique with newborn fathers participating in the entire breast massage process.

Nurses with > 5 years of clinical experience who had passed the qualification assessment provided systematic training on IBM to newborn fathers and fathers were required to pass a formal assessment before performing the massage on the mothers.

Prior to the massage, mothers were placed in a supine position with the head of the bed elevated at 45°. Massage pressure was applied only to the skin and superficial fascia layer without touching the underlying muscles or bones and the pressure was exerted collaboratively through the massager’s hands, forearms, and shoulders. The breast massage was conducted twice a day for 30 min each time until mother-infant reunion.

#### 2.3.3 Integrated breast massage technique

**Figure 1:**
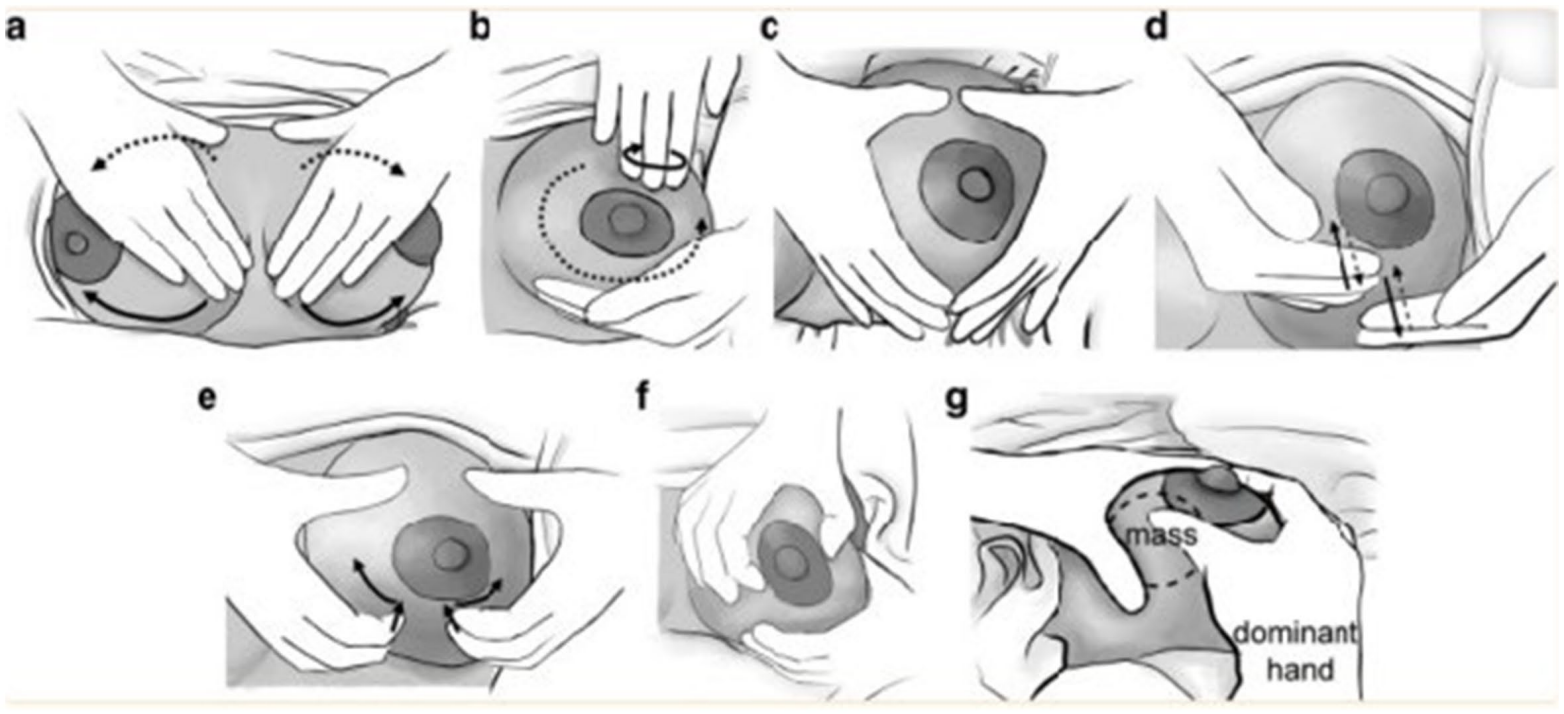
Schematic diagram of integrated breast massage (IBM) techniques. **(a)** Butterfly stroke: Press and move from the medial breast base to the axilla, repeat on the upper breast. **(b)** Fingertip circle: Gently knead the breast in a circular motion around the areola with one hand’s finger pads. **(c)** Diamond stroke (positioning): Clamp the breast in a diamond shape with both hands, move hands toward each other and the areola. **(d)** Diamond stroke (patting): Alternate hands to pat the breast with the palmar side of 2nd–5th fingers (press and release). **(e)** Diamond stroke (scratching): Press the breast with 2nd–5th finger tips, spread hands outward in a scratching motion, repeat toward the axilla around the breast. **(f)** Milk duct dilatation: Gently squeeze and roll the areola between the thumb and index finger. **(g)** Plugged duct drainage: Fix and squeeze breast lumps with the non-dominant hand; express milk by pressing the areola and nipple with the dominant thumb and index finger.

Pictorial images of the integrated breast massage technique show the massage therapist standing behind the patient’s head (Figure 1). A. butterfly stroke: apply continuous pressure to the affected breast while moving along the base of the breast from the medial side to the axillary area. Then repeat the same motion at the upper half of the breast. B. fingertip circle: with the fingers of one hand, lightly press and massage the breast in a circular motion while moving around the areola. C, D, E Diamond stroke. C: The breast is positioned between the fingers and thumbs of both hands (resembling a diamond shape), followed by both hands moving toward each other and toward the areola. D: Alternating between hands, press the breast with the palmar side of the 2nd to 5th fingers, and then release (resembling patting on the breast). E: With the tips of the 2^nd^ to 5^th^ fingers, press into the breast gently, then move both hands away from each other with an action that is similar to scratching. Repeat the same motions around the breast toward the axillary area. F Promotion of milk duct dilatation: Gently squeeze and roll the areolar area between the thumb and forefinger. G Augmentation of milk drainage from areas with plugged milk ducts: Gently fix and squeeze the breast mass with the non-dominant hand while the dominant hand manually expresses breast milk by gently compressing the areola and nipple between the thumb and index finger (15).

### 2.4 Measures

A survey was administered using the general information questionnaire, the Breastfeeding Self-efficacy Scale, the Breastfeeding Knowledge Questionnaire, the Social Support Scale, and the Postnatal Depression Scale. The primary outcome measures were the exclusive breastfeeding rates and feeding patterns in the two groups of mothers with all other outcomes defined as secondary. Data were collected before the intervention, 1 month after the intervention, and 3 months after the intervention.

#### 2.4.1 The general information questionnaire included

Maternal socio-demographic information, including age, occupation, education level, personal monthly income level, length of parental leave; Maternity history: mode of delivery, newborn weight, parental leave status, labor, pregnancy, duration of mother-infant separation; Basic information about the partner: ethnicity, occupation, education level and availability of parental leave.

#### 2.4.2 Scale

##### 2.4.2.1 Breastfeeding self-efficacy scale

This scale was developed in 1999 by Dennis and Faux as a tool to help mothers measure and self-report their confidence in breastfeeding; it consists of two dimensions, skill and inner activity, totaling 33 items (26). According to Bandura’s recommendations, all items are positive and scores are summed to yield a range from 33 to 165, with higher scores indicating higher levels of breastfeeding self-efficacy (27). In 2003, a Chinese scholar, Xiaona Dai (28), translated the Breastfeeding Self-Efficacy Scale into Chinese and validated its psychometric characteristics. The results showed that the Cronbach’s alpha coefficient of the Chinese version of the Breastfeeding Self-Efficacy Scale was 0.93, and deletion of any item would not increase it by more than 0.10.

##### 2.4.2.2 Breastfeeding knowledge questionnaire

This questionnaire was developed in Chinese by Zhao Min (29) and includes two dimensions, breastfeeding benefits and breastfeeding skills, with 11 entries related to breastfeeding benefits and 6 entries related to breastfeeding skills, totaling 17 entries. The content validity index of the questionnaire was 0.91, and the scoring method awards one point for a correct answer, and no points for a wrong or unclear answer. The total score ranges from 0 to 17, with higher scores indicating higher levels of breastfeeding knowledge, which can indirectly reflect the relationship between the level of breastfeeding knowledge and breastfeeding self-efficacy.

##### 2.4.2.3 Social support rating scale

This scale was developed in Chinese by Xiao Shuiyuan in 1994 (30). Based on the principles of validity and simplicity, and with reference to domestic and international studies, the scale was set-up with three dimensions: objective support, subjective support and utilization of social support, with three entries for objective support (2, 6, and 7), four for subjective support (1, 3, 4, and 5) and three for the utilization of social support (8, 9, and 10), for a total of 10 entries The scale is widely used in China with Cronbach’s alpha coefficients ranging from 0.825 to 0.896, and a retest reliability of 0.92. The scoring rules are as follows: answers to the entries (1, 2, 3, 4, 8, 9, 10) can only be selected as one item, and the answers are scored as 1–4 points according to the order of the options 1–4, respectively. Entries A, B, C, and D in entry 5 are scored as 1–4 points from none to full support, respectively; entry 7 is scored as 0 points if the answer is selected as without any source, and answers to the remaining items are scored as 0 points for any source, and several points for various sources of support. The higher the score, the higher the social support.

##### 2.4.2.4 Postpartum depression scale

The scale was designed at the University of Edinburgh in the United Kingdom and consists of 10 items to identify the psychological state of mothers who may have experienced postpartum depression in the past 7 days (31). The translated version from the Chinese University of Hong Kong is widely used in China, and the entries are rated on a 5-point scale (0, 1, 2, 3, 4), with a total score of 0 to 30, with higher scores indicating lower mood in the mother (32). The Chinese reliability coefficient of the scale was 0.87.

#### 2.4.3 Sample size calculation and statistical analysis

All mothers in the study joined a common WeChat group and data were collected by the researchers through this group and via telephone calls. Data were collected before the intervention, 1 month after the intervention and 3 months after the intervention.

The sample size was calculated using the formula for count data as the primary outcome indicator (33):


$$n=\frac{p_{1}(1-p_{1})+p_{2}(1-p_{2})}{{\left(p_{1}-p_{2}\right)}^{2}}\times {\left(\mu_{\alpha /2}+\mu_{\beta }\right)}^{2}$$

The research team determined that the 6-month exclusive breastfeeding rate among mother-infant separated mothers in our hospital was approximately 26.8%. The World Health Organization (WHO) has set a core global target of achieving a 60% 6-month exclusive breastfeeding rate by 3,030 (34). Here, *p_1_* represents the 6-month exclusive breastfeeding rate of mother-infant separated mothers in our hospital and *p_2_* denotes the target 6-month exclusive breastfeeding rate of 60% for this population. A relatively large sample size was adopted to facilitate subsequent evaluation of intervention effects and promotion of the research outcomes. With a two-sided significance level (*α*) set at 0.05, a type II error rate (*β*) at 0.1, and a 20% anticipated sample loss rate, the calculation indicated that a total of 106 mother-infant separated mothers (53 in the control group and 53 in the intervention group) were required for the 2 groups combined. Ultimately, a total of 120 mother-infant separated mothers were enrolled in this study.

The 6-month rate was used for sample size calculation based on WHO recommendations. The present study observed outcomes up to 3 months; long-term effects up to 6 months will be verified in future research.

IBM SPSS Statistics (version 22.0) was applied to analyze the data, the chi-square test was used to compare the rates of categorical variables, Student’s *t*-test was used to analyze data that conformed to the normal distribution and homogeneity of variance, for continuous variables that are normally distributed with repeated measurements at multiple time points, repeated measures ANOVA was used, expressed as mean ± standard deviation (^−^x ± s); for continuous variables that did not conform to the normal distribution or homogeneity of variance, the Wilcoxon signed-rank test (a non-parametric test for paired samples) was adopted for intragroup comparison and the Mann–Whitney U test (a non-parametric test for two independent samples) was used for intergroup comparison, and the data are expressed as the median and the upper and lower quartiles (M(*P*25, *P*75)). *p* < 0.05 indicates statistical significance.

## 3 Results

**Figure 2:**
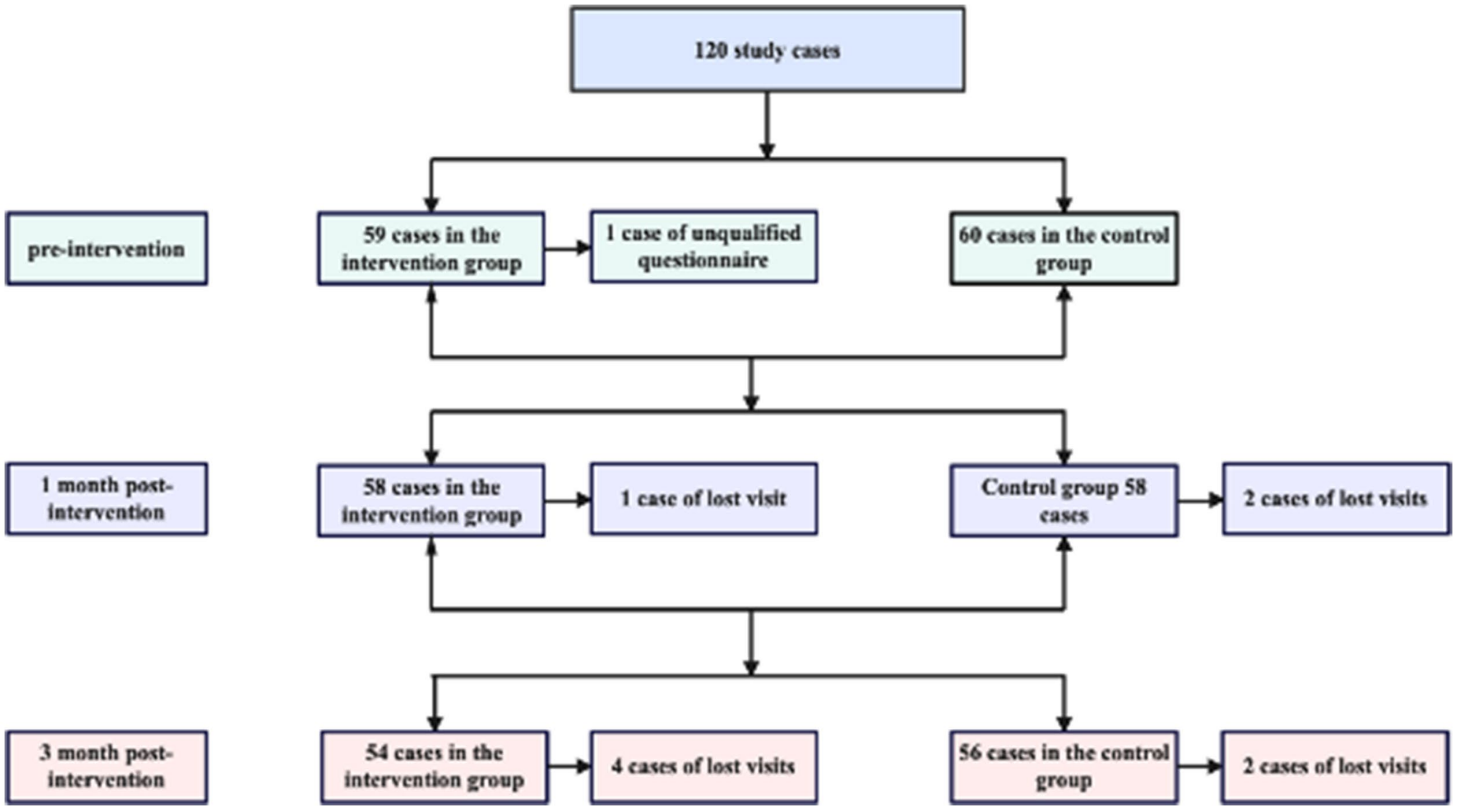
Flowchart of participant enrollment, follow-up, and loss to follow-up.

There were a total of 120 study participants, with 59 valid returned questionnaires in the intervention group and 60 in the control group; 58 valid returned questionnaires in the intervention group and 58 in the control group at 1 month post-intervention; and 54 valid returned questionnaires in the intervention group and 56 in the control group at 3 months post-intervention (Figure 2).

### 3.1 Comparison of baseline characteristics

Baseline matching was adopted during grouping and there was no significant difference in all sociodemographic characteristics, obstetric history, partner-related, newborn-related indicators, and scale scores between the two groups all *p* > 0.05, indicating good group balance. See Table 1 for details.

**Table 1:** Comparison of baseline characteristics between two groups.

	Intervention group, N(%; *n* = 60)/(^−^x ± s)	Control group, N(%; *n* = 60)/(^−^x ± s)	χ^2^/t	Cohen’s d/ η_p_2
Sociodemographic information^a^				
Age (years)				
<25	3 (5.1)	7 (11.7)		
25–29	18 (30.5)	15 (25.0)	3.408	−0.152
30–34	23 (39.0)	28 (46.7)		
>35	15 (25.4)	10 (16.7)		
Ethnic group				
Han ethnic group	59(100)	55(91.7)	3.271	−0.423
national minority	0	5(8.3)		
Educational attainment				
High school and below	8(13.6)	10(16.7)		
College or university	37(62.7)	42(70.0)	2.167	0.234
Master’s degree or above	14(23.7)	8(13.3)		
Current occupation				
Staff member	38(64.4)	39(65.0)		
Profession	10(16.9)	9(15.0)	4.216	−0.009
Others	11(18.7)	12(20.0)		
Average monthly income of individuals				
<5,000	16 (27.1)	14 (23.3)		
5,000–10,000	23 (39.0)	30 (50.0)	3.943	−0.348
10,000–30,000	18 (30.5)	16 (26.7)		
>30,000	2 (3.4)	0		
Obstetric history characteristics^b^				
The mode of delivery				
Vaginal delivery	17(28.8)	24(40.0)	1.648	0.236
Cesarean section	42(71.2)	36(60.0)		
Breastfeeding within 1 h				
Yes	49(83.1)	47(78.3)	0.425	−0.121
No	10(16.9)	13(21.7)		
Current breastfeeding practices				
Exclusive breast feeding	12(20.3)	14(23.3)		
Artificial feeding	10(16.9)	8(13.3)	0.381	0.029
Mixed feeding	37(62.7)	38(63.3)		
Maternal partners^c^				
Ethnic group				
Han ethnic group	58(98.3)	55(91.7)	1.527	−0.305
national minority	1(1.7)	5(8.3)		
Educational attainment				
High school and below	8(13.6)	11(18.3)		
College or university	38(64.4)	41(68.3)	1.770	0.230
Master’s degree or above	13(22.0)	8(13.4)		
Current occupation				
Staff member	48(81.4)	37(61.7)		
Profession	3(5.1)	7(11.7)	8.472	−0.350
Others	8(13.5)	16(22.6)		
Average monthly income of individuals				
<5,000	4(6.8)	4(6.7)		
5,000–10,000	22(37.3)	28(46.7)	1.526	0.102
10,000–30,000	28(47.5)	22(36.7)		
>30,000	5(8.5)	6(10)		
Availability of parental leave				
Yes	16(27.1)	20(33.3)	0.545	0.135
No	43(72.9)	40(66.7)		
Newborn information^d^				
Newborn birth weight	2910.59 ± 1099.115	2959.45 ± 893.202	−0.266	−0.049
Length of hospitalization of newborns (days)	17.85 ± 23.032	16.58 ± 20.933	0.313	0.058
Scale score^e^				
Total breastfeeding self-efficacy score	98.76 ± 23.438	99.2 ± 27.997	−0.092	−0.017
Total breastfeeding knowledge score	11.47 ± 3.919	12.07 ± 3.569	−0.862	−0.160
Social support scale total score	42.17 ± 5.960	40.97 ± 8.059	0.924	0.169
Depression scale total score	17.29 ± 5.446	16.45 ± 5.020	0.873	0.160

a Sociodemographic characteristics of parturient women, statistical analysis using chi-square test.

b Obstetric history characteristics of parturient women, statistical analysis using chi-square test.

c Demographic characteristics of maternal partners, statistical analysis using chi-square test.

d Newborn Information of two groups, statistical analysis using t-test.

e Baseline scores of the scales for the two groups, statistical analysis using t-test.

Baselines of the two groups are all *p* > 0.05.

Before the intervention, a baseline comparison of obstetric history characteristics between the two groups of parturient women showed that there was no significant difference in the mode of vaginal delivery, whether breastfeeding was initiated within 1 h, and the current method of breastfeeding, with all *p* > 0.05. See Table 1 for details.

Baseline comparison of the demographic characteristics of maternal partners in the two groups prior to the intervention, ethnicity, education, current occupation, average monthly personal income and the presence of parental leave, all gave *p* > 0.05. See Table 1 for details.

Before the intervention, there were no statistically significant differences between the two groups in terms of newborn birth weight, length of newborn hospital stay, total breastfeeding self-efficacy scores, total breastfeeding knowledge scores, total social support scale scores, and total depression scale scores (*p* > 0.05). See Table 1 and Table 1 for details.

### 3.2 Primary outcome

Analysis of the breastfeeding status in the two groups of parturient women at 1 month and 3 months post-intervention revealed significant differences in exclusive breastfeeding rates between the two groups at both 1 month and 3 months postpartum (*p* < 0.05). See Table 2 for details.

**Table 2:** Primary outcome measure.

Enterprise	Intervention group, N(%; *n* = 60)	Control group, N(%; *n* = 60)	χ^2^	Cohen’s d/ η_p_2
Breastfeeding				
1 month post-intervention				
Exclusive breast feeding	37(64.9)	13(22.4)		
Artificial feeding	2(3.5)	12(20.7)	23.068^**^	−0.686
Mixed feeding	18(31.6)	33(56.9)		
3 month post-intervention				
Exclusive breast feeding	36(64.3)	9(16.7)		
Artificial feeding	4(7.1)	18(33.3)	19.184^**^	−0.744
Mixed feeding	16(28.6)	27(50.0)		

*means *p* < 0.05, and **means *p* < 0.01.

### 3.3 Secondary outcome

**Figure 3:**
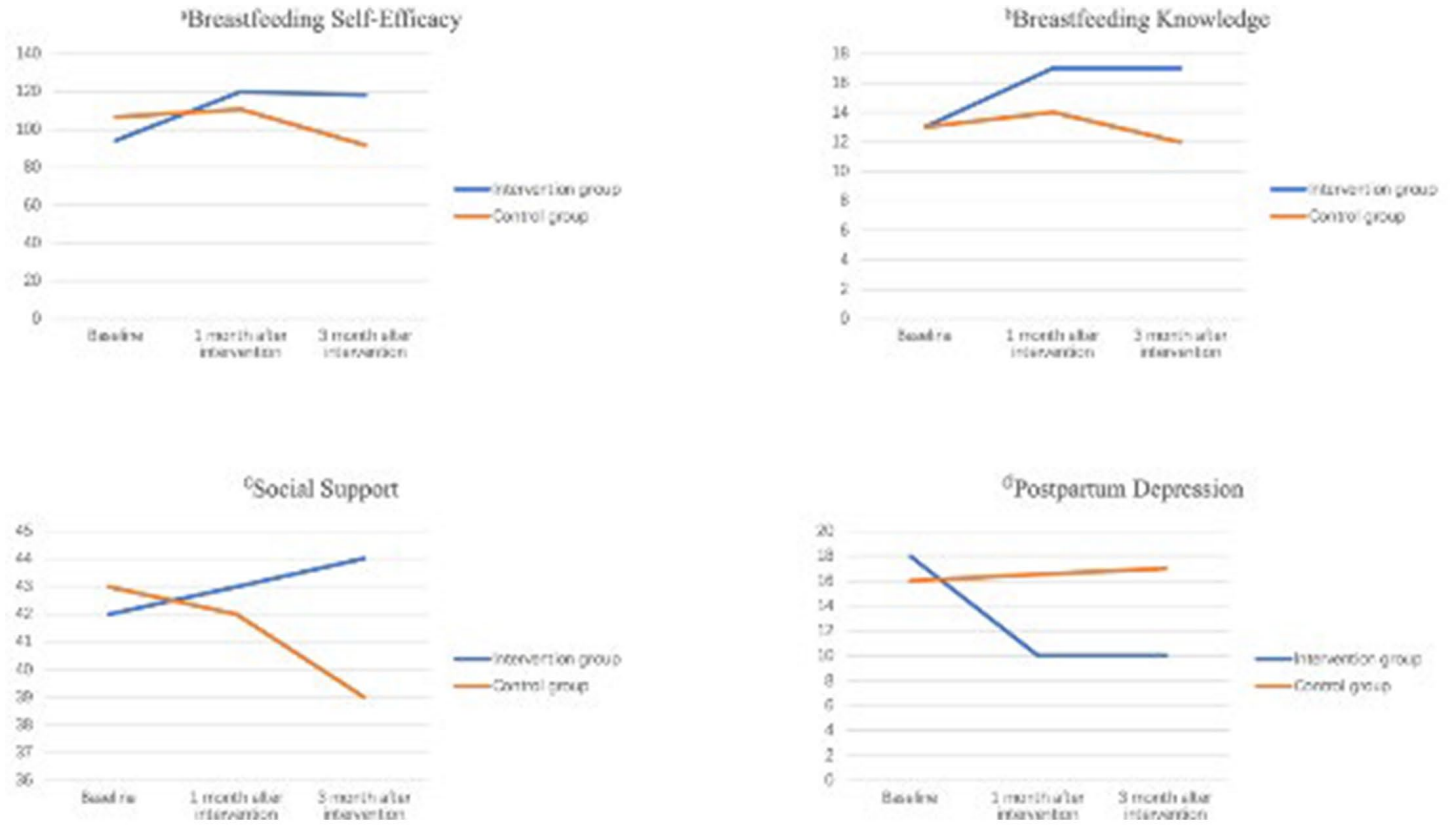
Trends in breastfeeding self-efficacy, breastfeeding knowledge, social support, and postpartum depression scores over time in the two groups.

Results for Breastfeeding Self-Efficacy: Repeated measures ANOVA on breastfeeding self-efficacy revealed that the following: The between-subjects main effect was significant [*F*(1, 98) = 16.33, *p* < 0.05, partial η^2^ = 0.14]. The main effect of measurement time was significant [*F*(2, 97) = 16.88, *p* < 0.05, partial η^2^ = 0.26]. The interaction effect between measurement time and group was significant [F(2, 97) = 3.92, *p* < 0.05, partial *η^2^* = 0.08]. The details are shown in Table 3 and Figure 3.

**Table 3:** Secondary outcome measure.

Enterprise	Point in time			F	*η^2^*
	Pre-intervention (^−^x ± s)	1 month post-intervention (^−^x ± s)	3 month post-intervention (^−^x ± s)		
Breastfeeding self-efficacy^a^					
Intervention group	98.76 ± 23.438	119.61 ± 12.034	112.54 ± 18.751		
Control subjects	99.20 ± 27.997	106.50 ± 20.123	91.73 ± 28.776		
t	−0.092	3.939**	4.362**		
Cohen’s d/ η_p_2	−0.017	0.719	0.796		
Between-subjects main effect				16.330^**^	0.143
Within-subjects main effect				16.879^**^	0.258
Interaction effect				3.924^*^	0.075
Breastfeeding knowledge questionnaire^b^					
Intervention group	11.47 ± 3.919	16.79 ± 1.221	15.57 ± 2.514		
Control subjects	12.07 ± 3.569	12.86 ± 3.431	10.68 ± 4.398		
t	−0.862	8.150**	6.997**		
Cohen’s d/ η_p_2	−0.157	1.488	1.278		
Between-subjects main effect				56.517^**^	0.366
Within-subjects main effect				23.480^**^	0.326
Interaction effect				14.713^**^	0.233
Social support rating scale^c^					
Intervention group	42.17 ± 5.960	43.23 ± 6.106	41.96 ± 7.004		
Control subjects	40.97 ± 8.059	41.43 ± 7.406	40.34 ± 7.380		
t	0.924	1.418	1.124		
Cohen’s d/ η_p_2	0.169	0.259	0.205		
Between-subjects main effect				0.852	0.009
Within-subjects main effect				2.042	0.040
Interaction effect				0.318	0.007
Postpartum depression scale^d^					
Intervention group	17.29 ± 5.446	13.19 ± 5.208	12.66 ± 4.859		
Control subjects	16.45 ± 5.020	16.69 ± 5.093	17.05 ± 4.885		
t	0.873	−3.640**	−4.469**		
Cohen’s d/ η_p_2	0.159	−0.665	−0.816		
Between-subjects main effect				19.013^**^	0.162
Within-subjects main effect				3.993^*^	0.076
Interaction effect				4.927^**^	0.092

a Breastfeeding self-efficacy score is the total score of breastfeeding self-efficacy scale.

b Breastfeeding knowledge questionnaire score is the total score of breastfeeding knowledge questionnaire.

c Social support rating scale score is the total score of social support rating scale.

d Postpartum depression scale score is the total score of postpartum depression scale.

*means *p* < 0.05, and **means *p* < 0.01.

Results for Breastfeeding Knowledge: Repeated measures ANOVA on breastfeeding knowledge revealed that the following: The between-subjects main effect was significant [*F*(1, 98) = 56.52, *p* < 0.05, partial *η^2^* = 0.37]. The main effect of measurement time was significant [*F*(2, 97) = 23.48, *p* < 0.05, partial *η^2^* = 0.33]. The interaction effect between measurement time and group was significant [F(2, 97) = 14.71, *p* < 0.05, partial *η^2^* = 0.23]. The details are shown in Table 3 and Figure 3.

Results for Social Support: Repeated measures ANOVA on social support revealed no statistically significant effects. Specifically, the between-subjects main effect was not significant [*F*(1, 98) = 0.85, *p* > 0.05, partial η^2^ = 0.01], the main effect of measurement time was not significant [*F*(2, 97) = 2.04, *p* > 0.05, partial η^2^ = 0.04], and the interaction between group and time was also not significant [F(2, 97) = 0.32, *p* > 0.05, partial η^2^ = 0.01]. These results indicate that social support levels did not differ significantly between the groups, change over time, or show different patterns of change across groups. The details are shown in Table 3 and Figure 3.

Results for Postpartum Depression: Repeated measures ANOVA on the postpartum depression scale scores showed the following. The main effect of measurement time was significant [F(2, 97) = 3.99, *p* < 0.05, partial η^2^ = 0.08]; The main effect of group was significant [F(1, 98) = 19.01, *p* < 0.05, partial η^2^ = 0.16]; The interaction effect between measurement time and group was significant [F(2, 97) = 4.93, *p* < 0.05, partial η^2^ = 0.09]. The details are shown in Table 3 and Figure 3.

## 4 Discussion

The results of this study showed that, 1 month after the intervention, the rate of exclusive breastfeeding in the intervention group was 64.9% (37 women), while the rate in the control group was 22.4% (13 women); 3 months after the intervention, the exclusive breastfeeding rate in the intervention group was 64.3% (36 women), and the rate in the control group at 3 months was 16.7% (9 women), with a significant difference between the two groups (*p* < 0.05). The exclusive breastfeeding rate in the intervention group of this study was higher than that in the control group, therefore it can be concluded that the intervention combining breast massage with shared parenting can improve the rate of exclusive breastfeeding for newborns. The exclusive breastfeeding rate in the intervention group was higher 3 months post-intervention than 1 month post-intervention. This is consistent with the conclusion that involving partners as members of the shared parenting team can improve their knowledge of breastfeeding, enhance their attitudes and involvement in breastfeeding, and increase the breastfeeding rate (35–37). Active co-parenting is defined as sharing responsibilities and goals, as well as teamwork between two people, working together to ensure the healthy growth of the child. Co-parenting in breastfeeding describes how parents share parenting tasks and cooperate to achieve breastfeeding goals (21). The research by Abbass-Dick and others found that an intervention based on the theory of shared parenting can effectively improve the breastfeeding rate, with significantly more mothers in the intervention group continuing to breastfeed at 12 weeks postpartum compared with the control group (96.2% vs. 87.6%, *p* = 0.02). Considering that breast massage can more quickly restore milk volume and increase the likelihood of a proper latch for the baby (12), the involvement of the partner in breast massage can improve the exclusive breastfeeding rate in mothers who have been separated from their infants.

Physiologically, the standardized integrated breast massage (IBM) (15) adopted in this study directly regulates lactation-related endocrine functions and local breast conditions: techniques including the butterfly stroke, fingertip circle and diamond stroke stimulate the breast, areola and axillary regions, activating sensory nerve endings that transmit signals to the hypothalamic–pituitary axis and inducing the pulsatile secretion of prolactin and oxytocin (12, 38), thereby enhancing the efficiency of milk synthesis and excretion. It also improves breast lymphatic and blood circulation (15), alleviates edema and distension, unblocks mammary ducts, and relieves breast discomfort, laying a physiological foundation for sustained lactation. (The exclusive breastfeeding rate of the intervention group reached 64.9% at 1 month and 64.3% at 3 months postpartum, which was significantly higher than the 22.4 and 16.7% of the control group.)

Psychosocially, paternal participation in breast massage as a co-parenting intervention transforms breastfeeding from a “solo task” into a “family responsibility,” enhancing maternal perceived emotional support and reducing feelings of loneliness and stress. Intimate interaction optimizes the spousal relationship (39) and reduces marital conflicts, while also boosting maternal breastfeeding confidence and strengthening the intrinsic motivation to persist in exclusive breastfeeding. The breastfeeding self-efficacy score of the intervention group increased from a baseline of 98.76 to 119.61 at 1 month postpartum. With positive feedback from joint participation, the Edinburgh Postnatal Depression Scale score of the intervention group decreased significantly from a baseline of 17.29 to 13.19 at 1 month and 12.66 at 3 months postpartum, whereas no obvious changes were observed in the relevant scores of the control group.

The positive cycle formed by physiological improvement and psychological support constitutes the fundamental reason why this intervention significantly increases the exclusive breastfeeding rate and improves maternal-related outcomes.

In this study the intervention combining breast massage with co-parenting significantly improved breastfeeding self-efficacy among mother-infant separated mothers with scores rising from a baseline of 98.76 to 119.61 at 1 month and 112.54 at 3 months post-intervention. These scores were significantly higher than the control group (106.50 at 1 month and 91.73 at 3 months, *p* < 0.05), a finding inconsistent with that of the Scott study (40). The core differences lie in the following aspects. The Scott study (40) implemented a single knowledge-based intervention for ordinary postpartum mothers during pregnancy, who had normal mother-infant interaction and few lactation issues, resulting in no significant intergroup difference. In contrast, the present study intervened immediately after childbirth targeting mother-infant separated mothers. This group had disrupted lactation feedback, lacked psychological support, and had extremely low initial breastfeeding confidence. In addition, instead of merely imparting knowledge, the intervention integrated breast massage (standardized operation by fathers) with co-parenting. Standardized massage by fathers can restore lactation feedback and improve lactation status, while paternal participation in co-parenting alleviates mothers’ loneliness and stress. These two aspects form a positive cycle. Even though breastfeeding confidence fluctuated slightly at 3 months, breastfeeding confidence remained far higher than the control group, achieving a rapid increase and long-term maintenance of breastfeeding confidence.

In this study breastfeeding knowledge scores showed an upward trend over time in both the intervention and control groups (intervention group: baseline 11.47, 1 month 16.79, 3 months 15.57; control group: baseline 12.07, 1 month 12.86, 3 months 10.68). This finding is consistent with the conclusion of relevant studies that “breastfeeding knowledge in both groups significantly increased compared with the baseline at 2 and 4 weeks postpartum (*P* < 0.05)” (40), which is presumably related to mothers independently acquiring feeding knowledge through various channels after childbirth. However, the knowledge scores of the intervention group at 1 and 3 months were significantly higher than the control group (t-values: 8.150 and 6.997, respectively, both *p* < 0.01). The core reason lies in the knowledge transmission mode during the intervention. The control group only passively received knowledge through routine care. During the process of fathers jointly participating in training and practical operations in the intervention group, the interactive communication between spouses further strengthened the mothers’ memory and understanding of the knowledge.

In this study there were no statistically significant differences in the Social Support Rating Scale scores between the two groups of puerpera at various time points (intervention group: baseline 42.17 ± 5.960, 1 month 43.23 ± 6.106, 3 months 41.96 ± 7.004; control group: baseline 40.97 ± 8.059, 1 month 41.43 ± 7.406, 3 months 40.34 ± 7.380; between-group main effect *F* = 0.852, *p* > 0.05). This neutral result is not due to the insufficient sensitivity of the scale to the specific breastfeeding support provided by partners but rather the combined effect of multiple objective factors and it holds equal research value to positive results. The Social Support Rating Scale used in this study has good reliability and validity [Cronbach’s *α* coefficient: 0.825–0.896; test–retest reliability: 0.92] (30), which can comprehensively measure the overall support obtained by the subjects from the entire social circle without measurement blind spots for partner-specific support. Furthermore, in the short term after childbirth, the core sources of social support for puerpera are family members and medical staff (36) and the overall support network is stable. Both the intervention and control groups received standardized routine medical and nursing care (25) with no baseline differences in partner support. Fathers’ participation in breast massage and co-parenting only optimizes the form of partner support, rather than essentially improving the overall social support level of puerpera. In addition, the intervention in this study is not aimed at enhancing overall social support but focuses on optimizing the pertinence and effectiveness of partner support in the context of breastfeeding (24). The effects are ultimately reflected in positive outcomes, such as improved breastfeeding self-efficacy and reduced postpartum depression (38), rather than changes in the total score of the Social Support Rating Scale. This neutral result accurately defines the boundary and pathway of the intervention effect, clarifying that the intervention exerts a role by activating the practical effectiveness of the existing support system in the breastfeeding context, rather than expanding the social support network (39). Complementary to positive results, it comprehensively interprets the multi-dimensional effects of the intervention and also provides important reference for the construction of a “comprehensive scale + special indicators” multi-evaluation system in future lactation support research and the optimization of clinical precise lactation support programs (24).

The clinical implementation of this intervention should be led by obstetric nurses/lactation consultants, adopting a nurse-led and father-participated model. The intervention shall be initiated within 24 h after childbirth for mothers separated from their newborns. First, one-on-one hands-on training and assessment on IBM will be provided for newborns’ fathers, who can only perform the massage for mothers upon passing the assessment. Systematic guidance on breastfeeding knowledge and co-parenting skills will be delivered synchronously. During hospitalization and the postpartum period, continuous follow-up, and problem-solving will be conducted via WeChat, telephone calls, and home visits.

The potential obstacles to its promotion and implementation mainly fall into two categories. First, some fathers have low acceptance and willingness to participate in the intervention due to insufficient awareness of lactation involvement and feelings of awkwardness toward breast massage. Second, the shortage of professional lactation nurses in primary medical institutions is likely to result in non-standard implementation of the intervention, making it difficult to guarantee the effectiveness of the practice.

This study had several limitations. First, the study used a single-center design with data collected from a tertiary maternity hospital in Shanghai. The participants were predominantly local residents with relatively high socioeconomic status, which limits the external validity of the findings. As such, the results may not be directly generalizable to primary medical institutions, rural areas, or postpartum women with lower socioeconomic status in other regions. Second, no blinding was implemented. Participants in the intervention and control groups were fully aware of the interventions they received, which may introduce performance and subjective bias in self-reported outcome measures, such as breastfeeding self-efficacy and postpartum depression. Third, potential selection bias exists because only couples who “fully accepted” breast massage were included, excluding those with neutral or negative attitudes toward breast massage. Fourth, the follow-up period was relatively short (only 3 months post-intervention), failing to align with the WHO recommendation of 6 months of exclusive breastfeeding, thus precluding the verification of the long-term sustainable effects of the intervention on maternal and infant outcomes. Fifth, a certain degree of follow-up loss occurred. The number of valid questionnaires in the intervention group decreased from 59 at baseline to 54 at 3 months, while in the control group the number dropped from 60 to 56. Although the loss rate is within an acceptable range, the loss rate may still compromise the representativeness of the results. Sixth, most outcome indicators rely on subjective self-report scales, lacking objective measurement data, such as actual milk volume and infant milk intake, which may lead to discrepancies between the study results and real-world conditions. Finally, postpartum lactation is also influenced by factors, such as postpartum diet and sleep status, and these confounding variables may have impacted the study findings.

Based on the limitations and unsolved problems of this study, targeted directions for future research are proposed as follows. First, in accordance with WHO recommendations, extend the follow-up period to 6 months and longer postpartum to verify the long-term and sustainable effects of the intervention on maternal and infant outcomes. Second, conduct multi-center and cross-population studies that include postpartum women in rural areas with low socioeconomic status and from different countries to analyze the applicability and effect differences of the intervention across diverse groups and provide evidence for its popularization and implementation. Third, add objective outcome indicators, such as breast milk quality, infant growth and development, maternal sleep, and marital relationship quality, and comprehensively evaluate the overall impact of the intervention by combining with subjective scales. Fourth, carry out controlled studies to compare the effectiveness and cost-effectiveness of breast massage performed by fathers versus professional medical staff, clarifying the advantages and applicable scenarios of different implementers. Fifth, explore the combined application of this intervention with measures, such as postpartum dietary guidance and psychological counseling, and build a more comprehensive breastfeeding support system for mothers separated from their infants.

## 5 Conclusion

This study demonstrates that integrating partners into a structured breast massage protocol following mother-infant separation serves as an effective nutritional support intervention. The study significantly enhances exclusive breastfeeding rates a direct indicator of optimal infant nutrition, while concurrently improving key maternal determinants of feeding success, namely breastfeeding self-efficacy and knowledge. Notably, the intervention also contributed to a measurable reduction in postpartum depression, a factor closely linked to maternal well-being and sustained nutritional care. These findings position partner involvement not only as a familial responsibility but as a vital component of evidence-based, family-centered clinical nutrition practice. We therefore recommend that healthcare units formally incorporate partner education and participation into postpartum lactation support programs, thereby safeguarding infant nutritional status and promoting integrated family health during a critical developmental window.

## Data Availability

The original contributions presented in the study are included in the article/supplementary material, further inquiries can be directed to the corresponding authors.
